# Antioxidant Therapy in Pancreatitis

**DOI:** 10.3390/antiox10050657

**Published:** 2021-04-23

**Authors:** Lourdes Swentek, Dean Chung, Hirohito Ichii

**Affiliations:** Department of Surgery, University of California, Irvine, CA 92868, USA; lyrobles@hs.uci.edu (L.S.); deandc@hs.uci.edu (D.C.)

**Keywords:** acute pancreatitis, chronic pancreatitis, oxidative stress, antioxidants, Nrf2, islet auto transplantation

## Abstract

Pancreatitis is pathologic inflammation of the pancreas characterized by acinar cell destruction and oxidative stress. Repeated pancreatic insults can result in the development of chronic pancreatitis, characterized by irreversible fibrosis of the pancreas and many secondary sequelae, ultimately leading to the loss of this important organ. We review acute pancreatitis, chronic pancreatitis, and pancreatitis-related complications. We take a close look at the pathophysiology with a focus on oxidative stress and how it contributes to the complications of the disease. We also take a deep dive into the evolution and current status of advanced therapies for management including dietary modification, antioxidant supplementation, and nuclear factor erythroid-2-related factor 2-Kelch-like ECH-associated protein 1(Nrf2-keap1) pathway activation. In addition, we discuss the surgeries aimed at managing pain and preventing further endocrine dysfunction, such as total pancreatectomy with islet auto-transplantation.

## 1. Introduction

Acute and chronic pancreatitis are inflammatory conditions marked by oxidative stress and acinar cell destruction. Repeated insults can result in eventual fibrosis of the pancreas and a multitude of secondary sequelae, which may lead to the loss of pancreatic function [1]. Acute pancreatitis (AP) has increased in incidence in the last 10 years [2]. In patients treated in the United States (US), pancreatitis was the most frequent gastrointestinal discharge diagnosis in 2009 [3]. Symptoms are characterized by abdominal pain, nausea, and pain radiating to the back. Although the severe form is only present in 5–10% of all cases of AP, the mortality for severe AP can be up to 50% [4,5]. Gallstones and alcohol are by far the most common etiologies in adults of Western countries but idiopathic causes surpassed both etiologies in a study by Frey et al. [6]. 

Recent literature showed an alarming increase in the frequency of cases of chronic pancreatitis (CP) as well as an increase in costs to the US health care system [7,8]. Despite a wide variety of potential causes including environmental (alcohol and smoking), genetic, and anatomic, up to 30% of patients have no identifiable cause [9]. Moreover, the presence of CP is the strongest risk factor for pancreatic cancer [10] and results in an almost 50% mortality approximately 20 years after the first diagnosis [11]. 

Complications related to AP can be vast, including pseudocysts, necrosis, and infections, and can be potentially devastating with the inflammation leading to acute respiratory distress syndrome, multisystem organ failure, and even death [12]. Management is focused on early resuscitative therapy with fluids and supportive care. In CP, the complications are related to the continued destruction of the pancreas. Weight loss, malabsorption, and steatorrhea are related to the advanced stages of exocrine destruction, and brittle diabetes becomes a destructive sequela of the loss of endocrine function [13]. Abdominal pain is well known to be a characteristic and debilitating symptom of the disease. The progressive development of fibrosis and loss of pancreatic tissue with loss of ductal secretory patency results in acute inflammation involving intra-pancreatic nerves contributing to an abdominal pain that is both incapacitating and intense. These patients are often on high doses of narcotics and are labeled as drug seekers. They are often shunned from the medical community due to the complexity of the pain, the frequent nature to which they present to the hospital, and the relative useless treatments we have available. In fact, major depression is a common side effect of this disease: one study indicated that over 20% of the pancreatitis patients suffering from constant, severe pain reported using antidepressants to manage their depression concomitant to pain [14]. 

Management remains supportive with nutritional and enzymatic supplementation. Surgery has remained reactive, managing the sequela of the devastating disease. Complications can be broad but as the disease progresses the loss of exocrine and endocrine functions become apparent. 

This review aims to first briefly discuss our understanding of AP, CP, and pancreatitis-related complications. Moreover, we will look over downstream pathways of oxidative stress implicated in pancreatitis. Lastly, the evolved therapeutic approaches to combat the downstream effect of oxidative stress in pancreatitis will be explored. 

## 2. Inflammation and Oxidative Stress in the Pathogenesis of Pancreatitis 

### 2.1. Acute Pancreatitis

According to general consensus, AP is characterized by the fulfillment of at least two of the following three criteria: clinically identifiable upper abdominal pain, serum amylase or lipase three or more times higher than the upper limit of normal, and distinctive abdominal imaging findings [15]. Although the pathophysiology of AP has yet to be fully deciphered, studies on humans and experimental animals strongly suggest that AP ensues from the disruption of acinar cell homeostasis [16]. Repeated exposure to stressors can overwhelm the protective mechanism of the pancreas and prompt the premature activation of digestive enzymes (e.g., trypsin), which in turn initiate the inflammatory cascade that promotes the auto-digestion of pancreatic tissue.

There is ample evidence that supports the connection between AP and inflammation of acinar cells followed by infiltration of innate and derived inflammatory molecules [17]. Acinar cell injury activates Nuclear factor-kappa B (NF-κB), inducing transcription of pro-inflammatory mediators that recruit leukocytes into the pancreas [16]. When leukocytes, such as neutrophils, are attracted to the site of inflammation, they adhere to vascular endothelial cells and infiltrate pancreatic tissue [18]. These cells amplify the inflammatory cascade in the pancreas and accentuate pancreatic injury by releasing various chemokines and cytokines, such as interleukins 1 and 6 (IL-1 and IL-6). Experimental studies on rats indicate that administration of ghrelin facilitates a decrease in inflammatory infiltration of the pancreas and reduces the level of pro-inflammatory cytokines, leading to the resolution of experimental AP [19,20].

Another consequence of local inflammation in AP is the injury of the vascular system within the pancreas. When endothelial cells are activated and destabilized during inflammation, vascular permeability is increased, resulting in vascular abnormalities and activation of the coagulation cascade [21]. Enhanced coagulation contributes to the progression of AP and in some cases manifests clinically in patients with severe acute pancreatitis (SAP) as life-threatening thrombotic complications [22]. It has also been shown that activation of coagulation leads to stimulation of inflammatory mechanisms, especially through the activation of thrombin, a pro-inflammatory enzyme that plays an integral role in the clotting cascade [23]. This adds weight to the assertion that coagulation and inflammation act reciprocally through a positive feedback mechanism during the progression of AP [21].

Among the many other pathways involved in the disease, oxidative stress is one of the most significant [24]. Injury to pancreatic acinar cells results when the body’s natural expression of cytoprotective molecules is overwhelmed by the oxidative stress created from the disorder [25]. The exocrine pancreas receives neuronal and endocrine input that causes the release of inactive digestive precursors from acinar cells into the duct [26]. Data reported by Brookes et al. suggest that calcium signaling and reactive oxygen species go hand in hand, and as indicated in the title of their article “calcium, ATP, and ROS: a mitochondrial love-hate triangle”, there is a complex interplay between calcium and oxidative stress [27]. Well-controlled calcium homeostasis is integral to the neurohormonal stimulation that leads to exocrine secretion. When sustained elevations of calcium-dependent reactive oxygen species are generated in the acinar cell, apoptosis occurs [25].

### 2.2. Chronic Pancreatitis

CP is a progressive destruction of the pancreas that results from repetitive inflammation, which accompanies the fibrosis of its native endocrine and exocrine tissue. If not properly treated, exocrine and endocrine pancreatic insufficiency could lead to deleterious consequences such as malabsorption, diabetes, and an increased risk of pancreatic cancer. The progression of CP is clinically observable, starting with acinar cell dysfunction, followed by beta-cell dysfunction, and lastly, a decrease in alpha-cell function which signifies the end stage of the disease. In a study involving patients with pancreatic resections, the vulnerability of specific pancreatic cells to chronic, progressive stress was observed and quantified. Examining pancreatic volume in CP patients compared with healthy controls revealed a decrease in beta-cell content by 29% and an approximately 10-fold increase in the numbers of apoptotic acinar cells, while there was no discernible difference in alpha-cell content [28].

It is recognized that excessive extracellular matrix (ECM) production contributes to the pathological changes associated with many different diseases, such as liver, lung, kidney, and pancreas. The overproduction of ECM leads to fibrosis and causes progressive organ destruction as seen in patients with prolonged CP [29]. Interestingly, activation of pancreatic stellate cells (PSCs) has been widely acknowledged as a key antecedent of pancreatic fibrosis, which is an evident hallmark of CP [30]. Repetitive oxidative stress in the pancreas transforms the fat-storing PSCs into myofibroblast-like cells that are capable of producing ECM, chemokines, and adhesion molecules in response to inflammatory infiltration [31].

## 3. Nrf2-Keap1 Signaling Pathway

**Figure 1:**
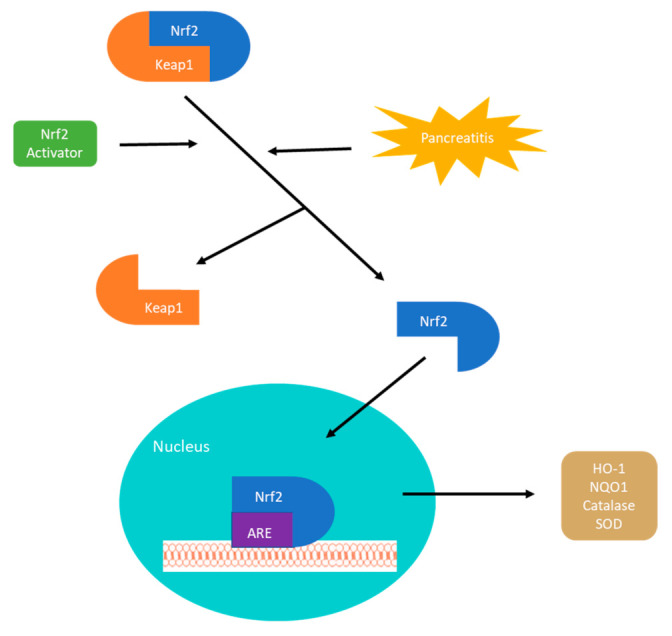
Nrf2-keap1 signaling pathway simplified diagram of the downstream effects of nuclear translocation. ARE, antioxidant response element; HO-1, heme oxygenase-1; Keap1, Kelch-like ECH-associated protein 1; NQO1, NAD(P)H: quinone oxidoreductase 1; Nrf2, nuclear factor erythroid 2-related factor 2; SOD, superoxide dismutase.

Oxidative stress is one of the most important mechanisms involved in the pathogenesis of AP and CP and finding a way to affect the front-line signals of inflammation remains the goal for many researchers. The Nrf2-keap1 (nuclear factor erythroid-2-related factor 2-Kelch-like ECH-associated protein 1) signaling pathway is the master regulator of the inflammatory response and exploiting this pathway has the capacity to create solutions for complex problems. Furthermore, dysregulation of the pathway has been implicated in several conditions including cancer, chronic disease, and aging [32]. The Nrf2-keap1 signaling pathway is the principal regulator of the anti-inflammatory and antioxidant response by mediating the transcription of genes encoding hundreds of antioxidant and detoxifying enzymes [33,34]. The Nrf2-Keap1 pathway plays a major role in the transcriptional regulation of inflammatory cytokines. It exists in the body as Nrf2, a transcription factor, and the repressor molecule Keap1. Under conditions of oxidative stress or in the presence of an Nrf2 activator, Nrf2 and Keap1 separate, and Nrf2 travels to the nucleus where it binds to the antioxidant response element gene. This will then result in the up-regulation of antioxidant genes and anti-inflammatory molecules [35,36] (Figure 1). Downstream target proteins of the pathway include heme oxygenase-1 (HO-1), NAD(P)H dehydrogenase, glutathione peroxidase 1, glutathione S-transferase (GST), glutathione reductase (GR), and superoxide dismutase (SOD) [37,38]. These antioxidant genes preserve the delicate cellular balance and maintain cellular homeostasis under stress and inflammation. Along with direct upregulation of genes, it also increased the synthesis of NADPH which in turn is a direct antioxidant and cofactor for many redox reactions [39,40]. This pathway is far more complex than the diagram below but its role in fighting oxidative stress is clear.

## 4. Roles of Antioxidant Therapy in Pancreatitis

### 4.1. Pharmacological Intervention

It has been reported that the levels of several antioxidants were deficient in many forms of oxidative stress. In a study conducted in Belgium, researchers found that patients with alcoholic CP were deficient in several blood levels of antioxidants despite controlling for dietary intake [35]. Polyphenols and phenols in olive oil have revealed excellent antioxidant activity [41]. Hydroxytyrosol (HT) is an antioxidant phenol with free radical scavenging activities and activates the Nrf2 pathway [42,43,44]. In an animal study by Fusco et al., HT was able to protect against pancreatitis and showed reduced inflammatory cytokines in both pancreas and colon tissue [45]. There was also decreased lipid peroxidation and oxidative stress overall. Numerous animal models have shown the benefits of antioxidant treatment on AP with a reduction in oxidative stress and inflammatory cytokines [46,47,48,49].

In human trials, data remain sparse and the conclusions continue to be elusive. In a small, randomized trial looking into the use of antioxidants for patients with CP, there was a significant improvement in pain in those taking antioxidants compared to placebo. Daily doses of 600 μg organic selenium, 0.54 g ascorbic acid, 9000 IU β-carotene, 270 IU α-tocopherol, and 2 g methionine showed a reduction in daily narcotic use and a reduction in painful days per month [50]. In a similar randomized double-blind trial, participants received either a placebo or exogenous antioxidant supplementation, namely Selenium, Ascorbic acid, and N-Acetylcysteine, for 6 months. The key findings of the study were that although there was an improvement in antioxidant levels in the supplemented patients, this intervention did not result in improvement in organ dysfunction, clinical outcomes, or reduced length of stay [51].

Gu et al. performed a meta-analysis of randomized controlled trials of antioxidants for the prevention of post ERCP pancreatitis. Though more than 3000 patients in 11 different studies were involved in the analysis, there did not appear to be a benefit in supplementation for prevention [52]. In another meta-analysis including eight studies patients with CP who received antioxidant therapy had a significant reduction in pain compared with the control group, including in a subgroup analysis of alcoholic and/or older patients [53]. Unfortunately, few of the trials were sufficiently powered and most were conducted at a single institution. The efficacy of naturally occurring antioxidants in the treatment of pancreatitis is yet to be determined [54]. However, benefits to antioxidant supplementation in the most infamous inflammatory disease raging over the world have been recently reported. Vitamin supplementation has been having a good year due to the overwhelming evidence that patients with COVID-19 have a significant depletion of antioxidants likely from increased utilization in counterbalancing free radicals [36]. In a recent review, due to strong combined evidence and international expert opinion, supplementation with vitamins in the battle against this global pandemic was a reasonable strategy [55]. That is a bold statement, especially since there have been so few therapies that have proven to help in this disease.

Rhubarb (*Rheum rhabarbarum*) is a species of perennial plant from the dried root and rhizome exerting its medicinal qualities. Over 200 compounds have been isolated from rhubarb [56]. Emodin is the most important active ingredient of rhubarb and has been used for the treatment of severe AP [57]. Researchers have shown that the therapeutic mechanism of emodin may be related to the reduction in DNA binding to NF-κB in pancreatic tissue with subsequent suppression of pro-inflammatory cytokines [58]. Apoptosis has been found to be a favorable response of the pancreas to injury and one of the mechanisms for emodin [59]. Emodin was injected in rats with AP and found a significant increase in apoptosis of pancreatic acinar cells [60]. Xiang et al. found that emodin inhibited the HTRA1/TGF-β/NF-κB signaling cascade [61]. It was also found to promote cell apoptosis via the calcium-mediated caspase-12 pathway and significantly attenuates calcium overload and decreases ER chaperone immunoglobin-binding protein [62,63,64,65,66]. In a rat model of severe AP, Jin and colleagues showed significant attenuation of AP as determined by histology and serum amylase through the inhibition of NF-κB, TNF-α, IL-6, IL1β [67]. Although no clinical trials have been done with emodin, there are numerous pre-clinical studies looking at the anti-cancer effects of the root [68,69,70].

Turmeric (*Curcuma longa*) is a yellow pigment derived from the rhizome, and it has been applied in traditional Chinese Medicine for a variety of diseases. There is a wide array of biological activities attributed to curcumin including anti-fibrosis, anti-apoptosis, anti-cancer, and anti-inflammation [71]. In a rat pancreatitis model, curcumin was able to reduce disease severity and showed a decreased activation of NF-κB as well as a reduction in mRNA induction of Il-6, TNF α, and iNOS in the pancreas [72]. Likewise, curcumin was able to reduce the severity of AP in a separate animal model through the Mitogen-activated protein kinase (MARK) signaling pathway [73].

There have also been multiple human studies using curcumin for a wide variety of applications with positive outcomes. The versatile spice has been studied in humans for its anti-inflammatory, neuroprotective, antioxidant, anti-proliferative, and anti-diabetic properties [74,75,76]. The poor bioavailability in certain studies has been addressed by using higher concentrations without causing toxicity [77]. In 2005, patients with tropical pancreatitis were treated orally with curcumin (500 mg) and piperine (5 mg) for 6 weeks and found a reduction in erythrocyte Malondialdehyde (MDA) levels and an increase in Glutathione compared with placebo [78].

Exploiting the Nrf2-Keap1 signaling pathway to combat pancreatitis has been a heavily researched topic. We observe through a culmination of various studies the remarkable ability for animals to sustain and neutralize extreme inflammation and oxidative stress under the presence of an Nrf2 activator [79,80]. In an experiment using human pancreatic tissue, an Nrf2 activator was able to produce higher expression of antioxidant enzymes, lower expression of inflammatory mediators, and greater cell viability against oxidative stress [81]. Kojayan et al. published an interesting review of CP and the treatment options related to a reduction in oxidative stress through antioxidants and Nrf2 activation. The Nrf2-keap1 signaling pathway was a paramount strategy against chronic inflammation [82]. In a rat pancreatitis model, we reported that a potent Nrf2 activator, Dimethyl Fumarate (DMF) was able to prove in vitro upregulation of antioxidants and in vivo showed a remarkable ability to significantly reduce inflammation and pancreatic destruction compared to controls [79]. Zhang et al. demonstrated long-term DMF treatment significantly resulted in improvement in beta-cell function in rats with CP [83]. Likewise, DMF was examined for its ability to protect exocrine and endocrine tissue in rats treated with 7 weeks of CP. The study showed atrophy of the pancreas, architectural damage, and elevated levels of oxidative stress in the control rats compared to those feed DMF [80]. It should be mentioned that, though the potency of DMF in preventing acinar destruction was identified in multiple rat pancreatitis models, there have been no human clinical trials using Nrf2 activators to treat AP or CP as of yet. On a separate note, DMF has been approved by the Food and Drug Administration for the purpose of treating psoriasis [84] and preventing relapse of multiple sclerosis [85].

### 4.2. Pain Control

It would be difficult to have a review regarding pancreatitis without the discussion of pain. The pain related to acute and chronic pancreatitis is complex and difficult to manage. It is clear the pain is not just a somatic manifestation but a complex neurohormonal pathology. The pancreas possesses extrinsic and intrinsic innervation. Extrinsic innervation stems from the vagus and splanchnic nerves, which carry sensory nerves from the dorsal root ganglia and sympathetic fibers from the ganglia of the sympathetic chain. Fibers of the vagus enter the pancreas or run through the celiac trunk to synapse on the organ’s intrinsic ganglia. Intrinsic innervation is made of intrapancreatic ganglia and is located on the islets [86]. Interestingly, the chronic inflammation related to pancreatitis results in neuroplastic alterations. We see increased tissue innervation and neuropathic alterations with nerve inflammation and neural invasion [87,88]. The addition of these facts results in a complex web of nerves that under inflammatory stress become larger and more complex resulting in further exacerbation of pain.

The management of patients’ pain is therefore complex. While a 3-step tiered approach to analgesia is recommended by the WHO, the complexity of the sensory nerves in the face of inflammation makes it clear that providing analgesia to patients with pancreatitis is like fixing a leaky faucet in a burning building. We mentioned in the previous paragraphs that several multi-centered trials have had success in managing pain with the use of antioxidants [53]. This approach honors and acknowledges that inflammation and oxidative stress are at the core of the pathophysiology of the patients’ pain.

Another interesting approach includes blocking the interweb of nerves surrounding the pancreas. A systematic review examined steroid-based endoscopic ultrasound-guided celiac plexus blocks, showing a satisfactory reduction of abdominal pain in 51% of patients [89]. Repeated treatments are usually necessary, and the overall data suggest a modest reduction in pain in some patients [90]. Surgical interventions aimed at regaining duct patency will prevent further ongoing inflammation and are discussed in the following chapter.

We have explored the role of Nrf2 activators in managing inflammation and quelling oxidative stress so their use in pain is an attractive topic. Zhou et al. created a rat model of Paclitaxel-induced neuroplastic pain and found that a potent Nrf2 activator was able to provide strong analgesic effects. The Nrf2 activator was found to delay the onset and in some cases completely abolish the pain. In the spinal cord of these rats, increased expression of HO-1 and Nrf2 was evident [91]. Although there are few studies examining this frontier, previous studies on the benefits of Nrf2 would suggest this would be an interesting subject matter for further consideration.

### 4.3. Surgical Approach

Medical therapy is widely recognized as a first-line approach in the management and treatment of patients with AP and CP. Various therapeutic modalities are employed, such as alcohol abstinence support, nutritional management (enteral feeding or parenteral feeding), analgesic agents, and antioxidants combined with pancreatic enzyme replacement therapy [92,93,94]. Nevertheless, an eventual cul-de-sac awaits many patients at the end of a long tunnel of medical therapy, owing to their ineffectiveness in stalling or reversing the progression of pancreatitis. Endoscopic therapies and surgical interventions, such as Endoscopic Pancreatic Necrosectomy, duodenum-preserving pancreatic head resection, Whipple procedure, the Puestow procedure, and total pancreatectomy with islet auto-transplantation (TPIAT) have emerged as feasible second-line treatments to address the sequela of CP [95,96,97,98]. All interventions aiming to restore ductal secretory patency and improve pain control. Among the interventions, TPIAT stands out from the rest due to the idiosyncratic and irreversible nature of the procedure, as well as the ability to address more than just pain. Pioneered by Sutherland and his colleagues at the University of Minnesota in 1977 for the treatment of CP, TPIAT has seen a gradual expansion in practice, and specific guideline for selecting patients eligible for TPIAT has also been established [94,99,100].

**Figure 2:**
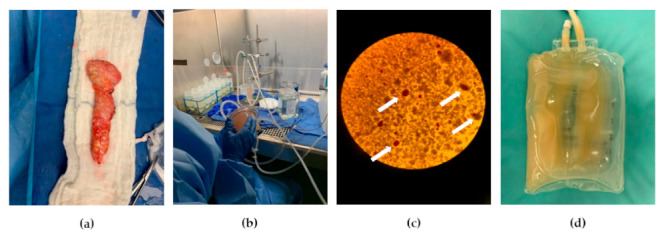
Total pancreatectomy with islet auto-transplantation (TPIAT): (**a**) Resected pancreas after removal of duodenum and vessels; (**b**) Enzymatic and mechanical digestion of the resected pancreas; (**c**) After collection of digested pancreatic tissue, samples are taken for microscopic inspection to assess islet size, number, and morphology with dithizone staining. White arrows indicate stained islet cells; (**d**) Digested pancreatic tissue with islets were transferred to an infusion bag for auto-transplantation.

The operative procedure of TPIAT, which lasts approximately 8–10 h, could be largely delineated in three steps. First, complete pancreatic resection is performed along with subsequent reconstruction of the GI tract. Enzymatic digestion of the resected pancreas and islet cell isolation ensues, followed by the infusion of islet cells into the portal vein and eventual engraftment into the liver (Figure 2) [94,101].

The rationale for performing TPIAT over total pancreatectomy (TP) alone is unambiguous. TP results in surgery-induced insulin-dependent diabetes mellitus, and islet auto-transplantation helps ameliorate the severity of such diabetes without any need for immunosuppression [102,103]. Therefore, the verdict on TPIAT largely depends on whether its efficacy over other second-line surgical interventions could be settled. Contemporary arguments against TPIAT frequently invoke the irreversibility and high expense of the procedure and a concomitant lifetime dependence on pancreatic enzyme replacement therapy (PERT). Moreover, the dearth of experienced medical centers that regularly perform TPIAT and the reluctance of insurance companies on covering the costs of islet isolation further substantiates the contention against embracing this modality to treat pancreatitis [94,104].

Even so, insulin-dependent diabetes and the uncontrolled destruction of islets that accompany chronic and recurrent AP warrant a second look into the desirability of TIPIAT. Rather than putting the patients through multiple repertoires of surgical procedures, none of which definitively culminates in a complete recovery or mitigation of intractable pain, TPIAT could be performed in advance once a differential diagnosis is made and eligibility is evaluated. Furthermore, the possibility of retrieving more functional islets and preventing opioid addiction which is interlocked with long-term pain management stimulates the assertion that an earlier TPIAT should be considered [94,104]. Over the past decade, multiple clinical studies on the efficacy and viability of TIPIAT have been performed in various medical centers across the United States [105,106,107,108,109,110,111,112,113,114,115,116]. These studies report, after their comprehensive preoperative and postoperative assessment, that the procedure yields a significant attenuation of insulin deficiency and a decrease in pain postoperatively. Postoperative enhancement in the patients’ quality of life as assessed using the Short Form (SF)-36 health questionnaire is also indicated in a subset of these studies [105,106,107,108,109,110,111,114,115,116] (Table 1). Among the researchers who conducted these studies, Bellin and her colleagues at the University of Minnesota are currently spearheading a 5-year multicenter study that aims to revamp conventional practice of TPIAT to improve pain relief, diabetes, and quality of life outcomes [117].

From a technical standpoint, two immediate challenges to the upscaled practice of TIPAT are islet yield and beta-cell survival, both of which are sine qua non for achieving complete insulin independence after total pancreatectomy. When TPIAT was first implemented to treat CP several decades ago, the outcomes were variable owing to the lack of standards for islet isolation. Nonetheless, continued research on islet isolation techniques since then has steadily improved the possibility of collecting high-quality islets from the resected pancreas [118,119,120,121].

Beta-cell survival is a more delicate issue, as transplanted islets are subject to apoptosis resulting from isolation stress and hypoxia. This is further aggravated by the instant blood-mediated inflammatory reaction, which is a prelude to the reduction of islet cells in the initial peri-transplant period [122,123]. Each step of the isolation process in fact leads to more oxidative stress and further loss of crucial cells.

In a study using human pancreatic tissue subjected to islet isolation, the vast differences between pancreatic alpha- and beta-cell were quantified in the face of oxidative stress. After isolation, the authors identified a reduction in the beta-cell to alpha-cell ratio, with a significant decrease in the number of beta-cells post-isolation. Moreover, beta-cells had a decreased expression of key anti-oxidants as compared to α-cells. In a viability assay, alpha-cells withstood the damage of oxidative stress and was less susceptible to oxidative stress-mediated DNA damage than beta-cells [124].

While beta-cell survival is an enigma left to be solved, up-to-date research using animal models and human islet cells has managed to shed light on novel drugs and transplant designs that can reasonably enhance beta-cell survival [125,126,127,128,129]. Masuda et al. showed how an Nrf2 activator was protective against beta-cell oxidative stress in human pancreatic tissue upon isolation [130]. The antioxidant effects of this Nrf2 activator gave vulnerable islets the resilience to withstand the stress of chronic induced inflammation and tissue isolation. Enhanced glucose tolerance in rats subjected to chronic inflammation was observed with activator supplementation. Likewise, islet viability in human pancreatic tissue in vitro was significantly improved when treated with an Nrf2 activator, DMF prior to isolation [80]. In another study, islet isolation was conducted in Nrf2 deficient mice to investigate the protective effects of Nrf2 on islet cells. The study illustrated improved islet yield and viability in the wild-type mice and even confirmed enhanced Nrf2 nuclear translocation, anti-oxidant expression, islet yield, and viability in rats treated with an Nrf2 activator (dh404) [131].

Interestingly, low molecular weight sulfated dextran (LMW-SD), which prevents the cascade that triggers an inflammatory reaction, was proven efficacious in promoting beta-cell survival in a recently completed phase II trial [132]. The drug Prolastin-C, an alpha-1 antitrypsin, is currently in phase I clinical trial and targets this inflammatory pathway to improve islet autograph survival and function in CP patients who have undergone TPIAT. Whether these trials strengthen the possibility of a comeback for TIPIAT is yet to be determined.

## 5. Discussion

Mukherjee et al. capture the reader in their excellent paper on precision medicine in AP by reciting the wisdom of our medical founding fathers. One of them is William Osler, who once famously remarked “If it were not for the great variability among individuals, medicine might as well be a science and not an art.” They then go on to remind you that despite international research we have yet to license targeted therapy for pancreatitis and have become less interested in discovering a therapy as well [133].

Given the increase in incidence and the frequent nature for which pancreatitis presents for emergency care, why are we studying it less? This very clever disease marked by a robust inflammatory response has managed to evade us and remain one step ahead of the scientists looking to stop it in its tracks. We currently have polarizing views on the benefits of supplementation of antioxidants in the treatment of disease. It would be worthwhile to solicit Osler himself for his thoughts on this matter, though his response might be that each and every one of us is unique, because what is deficient in one person might not be in another. Moreover, there are emerging questions regarding how much of each antioxidants to give, which antioxidants to give, and how much of the given antioxidants is absorbed in the body.

Examining the clinical trials that have explored the use of antioxidants in patients with AP or CP, the treatment algorithms are vastly different [134]. For example, using a combination of antioxidants (Selenium, vitamin C, β-carotene, and α-tocopherol) for seven days, Bhardwaj et al. were able to show a significant decrease in painful days per month, decreased narcotic use, and decreased hospitalizations [50]. Using a similar combination of selenium, β-carotene, L-methionine, vitamins C and E for 10 weeks, however, showed no difference in pain or emotional functioning, energy, or mental health [135]. Xu et al. randomized patients to glutamine for a 10-day infusion starting on day 1 or day 5 found a significant decrease in infection, operative rate, mortality, hospitalization, and duration of multi-system organ failure [136]. In contrast, Fuentes-Orozco et al. gave glutamine in standard total parenteral nutrition infusion for 10 days and found no difference in hospital stay or mortality [137]. Interestingly, using only a single agent vitamin C given in an IV infusion for 5 days showed a decrease in hospitalization and prevention of disease deterioration compared to control [138].

Despite the high doses of anti-oxidants used in multiple studies, the rate of complications was low, and the ones indicated were nausea, headache, and constipation [50,135]. Allopurinol administration included complications of malaise, headache, vomiting, and abdominal pain [139]. Although we have not found out the exact combination or timing, it does appear that the studies remained safe with few complications being described.

In spite of sufficient evidence in basic science literature for the role of antioxidants in pancreatitis, we have failed so far to translate the bench-top to the patient’s bedside. Perhaps what sets us apart from the animals studied in the laboratories may be the variability of our response to inflammation. For instance, it has been shown that age, smoking status, chronic alcoholism, nutritional status, and comorbidities play a critical role in patients’ response to inflammatory stressors and their early readmission [140]. While experimental subjects in the laboratory receive the same dose of an identical product in the absence of environmental or genetic differences, clinical environments can never be that tightly controlled. We can employ several exclusion criteria to maintain a modicum of consistency among the population being tested, but no human behaves in the same manner in the presence of stressors.

Perhaps humans have more control of their own fates and it is not a special pill that will fix everything but an actual way of life. Newer science, popular blogs, and health magazines are proposing that we could reverse inflammatory conditions with our diet.

*Fix it with Food* is a cookbook and tell-all of how one man essentially cured himself of his rheumatoid arthritis and external lupus by avoiding red meat, white flour, sugar, dairy, and alcohol [141]. At a first glance, depriving oneself of humankind’s most celebrated food products for the purpose of feeling better seems rather drastic. Or is it drastic that we would give ourselves a medicine to compromise our immune system to achieve the same or likely worse results?

The answer may not be far off from the idea that the food we nourish our body is, in fact, medicine. A diet focused on whole foods will provide the body with all the necessary antioxidants that it needs to run the efficient human machinery. We are also recognizing that the foods that we regularly consume are contributing to diseases and preventing us from protecting our body from inflammation and oxidative stress [142]. The idea that one or two antioxidants will make a difference in these patients for curbing or preventing disease is a naïve insult to the complexity of the human body in the face of inflammation. However, what if we influence the actual mechanism that controls all these molecules? As discussed previously, is the master regulator of all antioxidant molecules, Nrf2, a panacea for all inflammatory diseases?

Eastern medicine has already been using natural plant-derived Nrf2 activators long before researchers decided it had potent powers [143]. There use of plant-derived Nrf2 activators has been prescribed for a number of inflammatory conditions. Resveratrol, a naturally occurring Nrf2 activator, among other things, has been around for centuries, yet it has in recent years received some fanfare as there have been reports that this little compound is the fountain of youth. Improving and preventing age-related changes to the body and potent protective effects against oxidative stress [57].

The immune-modulatory properties of the Nrf2 activator, DMF, are potent and specific, unpredictably avoiding the secondary ill effects of systemic immunosuppression. The activity of the drug is complex, and the precise mechanism is not clear but the reduction in the inflammatory cytokine production and reduced inflammation seen in psoriasis plaques points to its effect on the main regulators of inflammation in the body. DMF also took a deep examination in its role in protecting MS patients in the study by Najjar et al. [144]. In the study, they illustrated DMF’s ability to promote regulatory B and T cells and noted reduced migration of immune cells. Active lesions of patients with MS are showered with IL-17 producing CD8+ cells and in patients labeled “DMF responders” there was a significantly lower frequency of IL-17 CD8+ cells, alluding to the suppression of these cells [145].

We have explored the remarkable ability of animals to sustain and neutralize extreme inflammation and oxidative stress under the presence of an Nrf2 activator. Proof of concept and in vitro and in vivo animal models are one thing, but the intricacy of the human body and the complexity of AP and CP has continued to elude us. The growing interest in Nrf2 activators to combat human disease makes this a worthwhile topic to explore in the context of pancreatitis. Our knowledge of the Nrf2 pathway has improved steadily over time, and evidence accrued in the laboratory each year has continued to support the use of Nrf2 activators for the treatment of AP and CP. Understanding this pathway can possibly be the key to unlocking viable treatment options for pancreatitis.

## 6. Conclusions

This review serves to provide the reader with up-to-date knowledge on AP and CP in the context of oxidative stress. We discuss treatment modalities such as antioxidant supplementation, dietary modification, and surgery. We then take a deep dive into the role of Nrf2 activators in neutralizing oxidative stress. Scientists are beginning to realize the necessity of working with the human body rather than against it. After all, the better we become at understanding and facilitating the cellular workings of our body, the more innovative we can get in improving our health. In the future, conducting a clinical trial that investigates the efficacy of Nrf2 activators in the prevention and treatment of AP and CP would help answer burning questions that currently occupy our minds.

## Figures and Tables

**Table 1:** Quality of life changes in TPIAT patients measured by SF-36 ^1.^

Source	Baseline					Post-TPIAT				
	*n*	BP	PCS	SF	MCS	*n*	BP	PCS	SF	MCS
Bellin et al. [106]	59	25	27	NR	30	65	53	36	NR	42
Bellin et al. [116]	19	24	30	25	34	19	79	50	80	46
Kotagal et al. [105]	11	NR	34	NR	NR	11	NR	55	NR	NR
Sutherland et al. [108]	160	22	29	32	38	66	49	38	60	49
Wilson et al. [110]	23	9	NR	32	NR	34	59	NR	76	NR
Wilson et al. [111]	15	7	NR	42	NR	17	65	NR	81	NR

BP, bodily pain; NR, not reported; MCS, mental composite score; PCS, physical composite score; SF, social functioning; TPIAT, total pancreatectomy with islet auto-transplantation. ^1^ *p* < 0.01 compared to baseline for all SF-36 scale scores. A higher BP score in SF-36 indicates less pain experienced by the patient.
